# Tonsillectomy does not reduce asthma in children: A longitudinal follow-up study using a national sample cohort

**DOI:** 10.1038/s41598-019-49825-3

**Published:** 2019-09-16

**Authors:** So Young Kim, Dong Jun Oh, Hyo Geun Choi

**Affiliations:** 1 Department of Otorhinolaryngology-Head & Neck Surgery, CHA Bundang Medical Center, CHA University, Seongnam, Korea; 2 Department of Internal medicine, Soonchunhyang University College, Seoul, Republic of Korea; 3 Department of Otorhinolaryngology-Head & Neck Surgery, Hallym University College of Medicine, Anyang, Korea; 4 Hallym Data Science Laboratory, Hallym University College of Medicine, Anyang, Republic of Korea

**Keywords:** Asthma, Risk factors

## Abstract

This study aimed to investigate the occurrence of tonsillectomy in asthmatic children using a control group with a comparable frequency of a preoperative history of asthma. Asthmatic children ≤15 years old were collected from the Korean Health Insurance Review and Assessment Service - National Sample Cohort (HIRA-NSC) from 2002 through 2013. In study I, asthmatic children who had undergone a tonsillectomy (n = 2,326) and control I participants (n = 9,304) were selected and matched 1:4 for age, sex, income, and region of residence but not a preoperative history of asthma. In study II, a preoperative history of asthma was additionally matched for between the tonsillectomy (n = 2,280) and the new control II participants (n = 9,120). The margin of equivalence of difference (control-tonsillectomy) for asthma was set at −0.05 to 0.05 per year. In addition, repeated measures ANOVA was performed for tonsillectomy according to yearly changes in asthma, status asthmaticus, and admission. In study I, the preoperative frequencies of asthma, status asthmaticus, and admission were higher in the tonsillectomy group than in the control group (P ≤ 0.001). The frequencies of postoperative asthma, status asthmaticus, and admission were lower in the tonsillectomy group than in the control I group for 3 years. In study II, the frequencies of postoperative 1-, 2-, and 3-year asthma and admission were not lower in the tonsillectomy group than in the control II group. Tonsillectomy did not further reduce the frequency of asthma in patients who underwent this procedure compared to the control group when a preoperative history of asthma history was equally matched between the two groups.

## Introduction

Asthma is one of the most common chronic diseases in pediatric and adult populations^1^. The prevalence of asthma is approximately 4.3% in adults in worldwide. In children, the prevalence of asthma has been estimated to vary from 2.8–31.2% according to ethnicity^2^. In Korea, approximately 10% of children suffer from asthma^3^. Allergic lower airway responses and inflammation induce airway hypersensitivity, airway remodeling, and asthma. The etiology of asthma is heterogeneous and influenced by multiple host-environmental interactions. In addition, probably due to the anatomical continuity of upper and lower airway, several studies suggested a systemic component of asthma^4,5^. The “united airway” hypothesis postulated that upper and lower airway diseases are comparably influenced by common inflammatory responses related to atopy^6^. In line with this, several previous studies have suggested that upper airway inflammation, for instance tonsillar hypertrophy, may be influence the development of asthma^7–9^. A recent systemic review reported a consistent correlation between tonsillectomy in asthmatic children and alleviated symptoms and parameters of asthma^7^.

Tonsillar hypertrophy is another common condition in children. Several factors, including upper airway inflammation and allergy, have been proposed as contributing factors for tonsillar hypertrophy. The rates of tonsillectomy are increasing^10,11^. Tonsillectomy is indicated to improve obstructive airway problems due to hypertrophic lymphoid tissue, infection or inflammation^12^. The effects of tonsillectomy for relieving inflammation might not be limited to the local upper airway inflammatory response^13^. A longitudinal observation study demonstrated decreases in the inflammatory marker chitinase in asthmatic children who underwent tonsillectomy^13^. Although the symptoms and aggravations of asthma were ameliorated after tonsillectomy, many studies were based on the poorly controlled asthmatic patients and limited to a small number of children^13,14^. Although one large population-based study with a matched control group for age, sex, and region of residence was conducted, that study did not consider patients’ preoperative history of asthma^15^. Thus, possible preoperative differences in asthma characteristics or severity could have resulted in differences in the occurrence of asthma after tonsillectomy probably due to the natural course of asthma recovery with age.

The running hypothesis of the present study was that the effects of tonsillectomy on asthma may have been overestimated in previous studies due to more severe preoperative asthma in tonsillectomy groups than in control groups. To prove this hypothesis, we designed two different studies with different control groups. In study I, the control group was matched for age, sex, income, and region of residence, as in previous studies. In addition to these demographic factors,a preoperative history of asthma was matched between the new control group and the study group in study II. In both studies, the impact of tonsillectomy on asthma was evaluated. The primary outcome was the frequency of asthma treatment after tonsillectomy. Moreover, the frequencies of status asthmaticus and admission related to asthma were analyzed as secondary outcomes. All asthma-related parameters were investigated for a follow-up periods up to 3 years.

## Results

Age, sex, income, and region of residence were matched between the tonsillectomy and control I and control II groups in both study I and study II (Table 1). There were differences in the preoperative and postoperative 1-, 2-, and 3-year asthma frequencies between the tonsillectomy and control I groups in study I (P < 0.001 of repeated measures ANOVA; Table 2). The frequency of preoperative asthma was higher in the tonsillectomy group than in the control I group (1.24 ± 2.91 for the tonsillectomy group vs. 0.93 ± 2.54 for the control I group, 95% CI of differences = −0.44 to −0. 20). The frequency of postoperative 1-year asthma was also higher in the tonsillectomy group than in the control I group (0.90 ± 2.91 for the tonsillectomy group vs. 0.73 ± 2.28 for the control I group, 95% CI of differences = − 0.30 to −0.04). The frequencies of postoperative 2- or 3-year asthma were comparable between the tonsillectomy and control I groups. For status asthmaticus, the frequency of postoperative 2-year status asthmaticus was lower in the tonsillectomy group than in the control I group (0.00 ± 0.06 for the tonsillectomy group vs. 0.01 ± 0.20 for the control I group, 95% CI of differences = 0.00 to 0.01; Table 3). Regarding admission history, the frequency of preoperative and postoperative 1- and 2-year admission were higher in the tonsillectomy group than in the control I group. Furthermore, the frequency of postoperative 3-year admission was comparable between the tonsillectomy and control I groups.

**Table 1:** General Characteristics of Participants in Study I and Study II (matched 1:4).

Characteristics	Study I (n = 11,630)		Study II (n = 11,400)	
	Tonsillectomy group (n, %)	Control I group (n, %)	Tonsillectomy group (n, %)	Control II group (n, %)
Age (years old)				
0–4	283 (12.2)	1,132 (12.2)	278 (12.2)	1,112 (12.2)
5–9	1,553 (66.8)	6,212 (66.8)	1,518 (66.6)	6,072 (66.6)
10–14	490 (21.1)	1,960 (21.1)	484 (21.2)	1,936 (21.2)
Sex				
Male	1,452 (62.4)	5,808 (62.4)	1,424 (62.5)	5,696 (62.5)
Female	874 (37.6)	3,496 (37.6)	856 (37.5)	3,424 (37.5)
Income				
1 (lowest)	7 (0.3)	28 (0.3)	7 (0.3)	28 (0.3)
2	83 (3.6)	332 (3.6)	81 (3.6)	324 (3.6)
3	93 (4.0)	372 (4.0)	87 (3.8)	348 (3.8)
4	104 (4.5)	416 (4.5)	99 (4.3)	396 (4.3)
5	130 (5.6)	520 (5.6)	126 (5.5)	504 (5.5)
6	179 (7.7)	716 (7.7)	177 (7.8)	708 (7.8)
7	246 (10.6)	984 (10.6)	241 (10.6)	964 (10.6)
8	313 (13.5)	1,252 (13.5)	306 (13.4)	1,224 (13.4)
9	427 (18.4)	1,708 (18.4)	420 (18.4)	1,680 (18.4)
10	402 (17.3)	1,608 (17.3)	399 (17.5)	1,596 (17.5)
11 (highest)	342 (14.7)	1,368 (14.7)	337 (14.8)	1,348 (14.8)
Region				
Urban	992 (42.6)	3,968 (42.6)	965 (42.3)	3,860 (42.3)
Rural	1,334 (57.4)	5,336 (57.4)	1,315 (57.7)	5,260 (57.7)

**Table 2:** Difference of mean values of pre-operative and post-operative asthma between tonsillectomy and control group.

	Tonsillectomy (mean, SD)	Control (mean, SD)	95% CI of difference	P-value of Independent T-test	P-value of repeated measured ANOVA
**Study I**					
Pre-op asthma	1.24 ± 2.91	0.93 ± 2.54	−0.44 to −0.20	<0.001*	<0.001*
Post-op 1 year asthma	0.90 ± 2.91	0.73 ± 2.28	−0.30 to −0.04	0.009*	
Post-op 2 year asthma	0.65 ± 2.17	0.63 ± 2.17	−0.12 to 0.08	0.700	
Post-op 3 year asthma	0.52 ± 2.44	0.51 ± 1.82	−0.10 to 0.08	0.887	
**Study II**					
Pre-op asthma	0.98 ± 1.89	0.98 ± 1.89	−0.09 to 0.09	1.000	0.577
Post-op 1 year asthma	0.81 ± 2.59	0.76 ± 2.29	−0.16 to 0.58	0.362	
Post-op 2 year asthma	0.59 ± 1.89	0.61 ± 1.90	−0.07 to 0.11	0.676	
Post-op 3 year asthma	0.47 ± 1.53	0.48 ± 1.65	−0.06 to 0.09	0.758	

^*^Statistical significance at P < 0.05.

SD: Standard deviation.

CI: Confidence interval.

Difference: Control group – Tonsillectomy group.

Repeated measured ANOVA using Greenhouse-Geisser correction.

**Table 3:** Difference of mean values of pre-operative and post-operative status asthmaticus and admission (ER visit or admission) between tonsillectomy and control group.

	Tonsillectomy (mean, SD)	Control (mean, SD)	95% CI of difference	P-value of Independent T-test	P-value of repeated measured ANOVA
**Study I**					
Pre-op Status asthmaticus	0.03 ± 0.46	0.01 ± 0.17	−0.04 to 0.00	0.054	0.001*
Post-op 1 year Status asthmaticus	0.01 ± 0.12	0.01 ± 0.21	−0.01 to 0.01	0.961	
Post-op 2 year Status asthmaticus	0.00 ± 0.06	0.01 ± 0.20	0.00 to 0.01	0.048*	
Post-op 3 year Status asthmaticus	0.00 ± 0.07	0.01 ± 0.17	0.00 to 0.01	0.060	
Pre-op admission	0.03 ± 0.21	0.01 ± 0.13	−0.03 to −0.01	<0.001*	<0.001*
Post-op 1 year admission	0.02 ± 0.20	0.01 ± 0.11	−0.02 to −0.00	0.026*	
Post-op 2 year admission	0.02 ± 0.17	0.01 ± 0.11	−0.01 to 0.00	0.045*	
Post-op 3 year admission	0.01 ± 0.14	0.01 ± 0.11	−0.01 to 0.01	0.758	
**Study II**					
Pre-op Status asthmaticus	0.02 ± 0.43	0.01 ± 0.20	−0.03 to 0.01	0.172	0.017*
Post-op 1 year Status asthmaticus	0.01 ± 0.12	0.01 ± 0.13	−0.01 to 0.01	0.770	
Post-op 2 year Status asthmaticus	0.00 ± 0.06	0.01 ± 0.21	0.00 to 0.01	0.028*	
Post-op 3 year Status asthmaticus	0.00 ± 0.06	0.01 ± 0.15	0.00 to 0.01	0.063	
Pre-op admission	0.03 ± 0.21	0.02 ± 0.16	−0.02 to −0.00	0.006*	0.037*
Post-op 1 year admission	0.02 ± 0.17	0.01 ± 0.10	−0.02 to −0.00	0.032*	
Post-op 2 year admission	0.02 ± 0.17	0.01 ± 0.11	−0.02 to 0.00	0.042*	
Post-op 3 year admission	0.01 ± 0.14	0.01 ± 0.10	−0.01 to 0.00	0.799	

^*^Statistical significance at P < 0.05.

SD: Standard deviation.

CI: Confidence interval.

Difference: Control group – Tonsillectomy group.

Repeated measured ANOVA using Greenhouse-Geisser correction.

In study II, the frequency of preoperative asthma was matched between tonsillectomy and control II group. The frequencies of postoperative 1-, 2-, and 3-year asthma were not different between the tonsillectomy and control II groups (P = 0.577 of repeated measures ANOVA; Table 2). For status asthmaticus history, the frequencies of preoperative and postoperative 1- and 3-year status asthmaticus did not differ between the tonsillectomy and control II groups, although the frequency of postoperative 2-year status asthmaticus was lower in the tonsillectomy group than in the control II group (0.00 ± 0.06 for the tonsillectomy group vs. 0.01 ± 0.21 for the control II group, 95% CI of differences = 0.00 to 0.01; Table 3). Regarding admission history, the frequencies of pre- and postoperative admission were lower in the control II group than in the tonsillectomy group (P = 0.037 of repeated measures ANOVA). In accordance with the frequency of preoperative asthma, the low, high, and very high preoperative asthma groups demonstrated comparable pre- and postoperative frequencies of asthma between the tonsillectomy and control II groups (Table 4 and S1 Table).

**Table 4:** Subgroup analysis of pre-operative and post-operative asthma between tonsillectomy and control group according to preoperative asthma frequency in Study II (Low Vs high pre-operative asthma group).

	Tonsillectomy (mean, SD)	Control (mean, SD)	95% CI of difference	P-value of Independent T-test	P-value of repeated measured ANOVA
**Low pre-operative asthma (<2 times a year, n = 8,880)**					
Pre-op asthma	0.18 ± 0.38	0.18 ± 0.38	−0.02 to 0.02	1.000	0.212
Post-op 1 year asthma	0.60 ± 1.71	0.54 ± 1.77	−0.15 to 0.03	0.188	
Post-op 2 year asthma	0.45 ± 1.51	0.49 ± 1.67	−0.05 to 0.12	0.437	
Post-op 3 year asthma	0.38 ± 1.31	0.40 ± 1.53	−0.07 to 0.09	0.770	
**High pre-operative asthma (≥2 times a year, n = 2,520)**					
Pre-op asthma	3.79 ± 2.36	3.79 ± 2.36	−0.23 to 0.23	1.000	0.993
Post-op 1 year asthma	1.55 ± 4.39	1.54 ± 3.45	−0.37 to 0.35	0.954	
Post-op 2 year asthma	1.08 ± 2.82	1.04 ± 2.50	−0.29 to 0.22	0.783	
Post-op 3 year asthma	0.75 ± 2.09	0.76 ± 1.99	−0.18 to 0.21	0.901	

^*^Statistical significance at P < 0.05.

SD: Standard deviation.

CI: Confidence interval.

Difference: Control group – Tonsillectomy group.

Repeated measured ANOVA using Greenhouse-Geisser correction.

## Discussion

Tonsillectomy did neither decrease nor increase the frequency of asthma treatment, status asthmaticus, or hospitalization in asthmatic children when the control group was matched for preoperative asthma history (control II) in the present study. In the subgroup analyses, the severity of asthma did not influence the relationship between tonsillectomy and asthma. Matching for the preoperative frequencies of asthma enabled an unbiased comparison between the tonsillectomy and control group in this study (study II, tonsillectomy vs. control II group). On the other hand, tonsillectomy reduced the frequency of asthma comparable to the control group after 2 years postoperatively when the control group was not matched for the preoperative frequency of asthma (study I, tonsillectomy vs. control I group).

Similar to our study I results, several previous studies have demonstrated relief of asthma after tonsillectomy using a control group unmatched for preoperative asthma history^13,15,16^. A large population-based study demonstrated that of asthmatic children who underwent tonsillectomy, 30.2% of patients with acute asthma exacerbation, 37.9% of patients with acute status asthmaticus, and 35.8% of patients with asthma-related hospitalization had reduced frequencies of these events 1 year after tonsillectomy compared to asthmatic children without tonsillectomy (95% CI = 25.6–34.3%, P < 0.001 for acute asthma exacerbation; 95% CI = 29.2–45.6%, P < 0.001 for acute status asthmaticus; 95% CI = 19.6–48.7%, P = 0.02 for asthma-related hospitalization)^15^. However, the asthma-related parameters were worse in the asthma group than in the control group in that study^15^. For instance, the frequencies of acute asthma exacerbation and acute status asthmaticus were 16.6% and 4.2%, respectively, in the asthma group and 12.6% and 3.1%, respectively, in the control group^15^. A longitudinal, observation study reported improvement in asthma control in children with poorly controlled asthma^13^. Another study also supported the effectiveness of tonsillectomy in poorly controlled asthmatic children^14^. Poorly controlled asthmatic children with obstructive sleep apnea had a decreased annual frequency of acute asthmatic exacerbations, rescue inhaler use, and asthma symptoms after tonsillectomy^14^. In other words, tonsillectomy might be effective in only a proportion of asthmatic children with poor control or severe asthma. Although most previous studies reported the reduction of asthma severity after tonsillectomy^7^, a retrospective case-control study suggested the increase the risk of asthma after tonsillectomy (odds ratio = 1.96, 95% CI = 1.14–3.36)^17^. However, that study did not match between tonsillectomy and control patients and conducted univariate analysis. A longitudinal birth cohort study demonstrated no association of the tonsillectomy in childhood with the adult asthma (relative risks = 0.93, 95% CI = 0.52–1.64)^18^. Therefore, the effect of tonsillectomy on asthma might not be considerable if control group exclude the effect of prior asthma.

Early-onset asthma is more likely to be associated with allergy than adult-onset asthma. Thus, the increased allergic response in hypertrophied tonsils has been suggested to aggravate asthma^19^. However, the relationship between tonsillar hypertrophy and allergic asthma has been controversial. A recent study suggested higher prevalences of sensitization to allergens, specific immunoglobulin E (IgE), and asthma in children with tonsillar hypertrophy than in participants in a control group^19^. On the other hand, a cross-sectional study demonstrated that tonsillar hypertrophy was not associated with atopy^20^. Likewise, another study reported that the levels of specific IgE were not elevated in tonsillectomy patients compared to participants in a control group^21^. The characteristics of the study and control groups could have resulted in these inconsistencies due to an association between tonsillar hypertrophy and asthma.

Several possible reasons could explain the lack of a definitive association between tonsillectomy and asthma when control group was matched for preoperative asthma histories (study II). First, the degeneration of hypertrophic tonsils together with the relief of asthma with age could have mitigated the impact of tonsillectomy in asthmatic children in this study. Only one-half to one-fourth of asthmatic children persisted to become adult asthmatics in a population-based, longitudinal cohort study^22,23^. Several characteristics were suggested to influence the persistence of asthma in adulthood including sensitization to house dust mites, airway hypersensitiveness, early onset of asthma and decreased lung function reflected by low forced expiratory volume (FEV1)^23^. In addition, obstruction of upper airway due to tonsillar hypertrophy is relieved with age. Therefore, the contribution of tonsillectomy to upper airway patency may be decreased with age. Indeed, a large proportion of children, approximately 77%, underwent tonsillectomy because of upper airway obstruction, which could spontaneously resolve with age^11^.

The bystander effects of common airway inflammation and atopic diseases could mediate the association between tonsillar hypertrophy and asthma when the preoperative asthma histories were not considered as in previous studies and our study I. Severe airway inflammation and atopic conditions may induce upper airway disorders in addition to lower airway disorders, which can be relieved after tonsillectomy. Although upper airway disorders were relieved, lower airway disorders might have remained after tonsillectomy in patients who underwent this procedure comparable to those who did not. It is possible that upper airway obstruction in children with hypertrophic tonsils might be misdiagnosed as lower airway disease such as asthma. A retrospective study demonstrated that the use of respiratory medication, which was a nonantimicrobial prescription for lower respiratory disorders including bronchitis, bronchiolitis, or asthma, was decreased approximately by 32% during the year after tonsillectomy^24^. That study concluded that similar respiratory symptoms of coughing, wheezing, and shortness of breath may have led to a misdiagnosis of upper airway disease as a lower respiratory disorder in these patients^24^.

Tonsillectomy may be effective only for relieving the systemic airway inflammation associated with asthma but not be sufficiently effective for reversing the disease course of asthma. Tonsillar hypertrophy could have different pathophysiologic mechanism than asthma. Local lymphoid tissue, such as tonsils, might be a source of inflammation that could trigger airway inflammation and remodeling in asthmatic patients. Indeed, numerous inflammatory cytokines and chemokines are highly expressed in adenoid tissue in asthmatic children^25^. However, only a small number of cytokines or chemokines, such as tumor necrosis factor-β, fibroblast growth factor-2, and growth-regulated oncogene, were increased in asthmatic children compared to nonasthmatic children^25^. Thus, it can be supposed that specific local inflammatory or allergic pathophysiology may be responsible for upper and lower airway diseases. In line with this finding, the inflammatory marker YKL-40, which is one of the major chitinases in humans, was not changed after tonsillectomy; however, a marker of systemic inflammation of chitinase activity was reduced^13^.

This study adds to previous findings by using a matched control II group for preoperative history of asthma. Moreover, a large, representative study population strengthened the reliability of the present results. Because HIRA data includes all citizens without exception, this study minimized selection bias. The sample cohort was selected to represent the mother population, as described in methods section, which was verified in a previous study^26^. The physician-diagnosed ICD 10 codes, rather than subjective questionnaires, were used to define both asthma and tonsillectomy.

However, a few limitations should be considered when interpreting the present results. The indications for tonsillectomy, such as obstructive or inflammatory symptoms, could not be defined in the present study group. It is possible that there were some variations in the study group probably due to the indications for tonsillectomy. In terms of asthma classification, the severity and treatment history of asthma could not be classified in this study. In addition, although a number of confounders were considered in this study, there was a lack of information on lifestyle factors including secondhand smoking, sleep, obesity, diet and nutrition in the NHIS data; thus, the possible confounding effects of these lifestyle factors on the present results could not be eliminated.

In conclusion, tonsillectomy did not decrease the frequency of asthma treatments and related hospitalizations in children. The higher preoperative burden of asthma in the tonsillectomy group than the control group who did not matched for the preoperative asthma histories could have resulted in greater changes in asthma frequency after tonsillectomy.

## Materials and Methods

### Study population and data collection

The ethics committee of Hallym University (2017-I102) approved the use of these data. The Institutional Review Board exempted the requirement for written informed consent. All methods were performed in accordance with the guidelines and regulations of the ethic committee of Hallym University.

This national cohort study used data from the Korean National Health Insurance Service-National Sample Cohort (NHIS-NSC). The detailed description of this data was described in our previous studies^27,28^.

### Participant selection

#### Study I

**Figure 1:**
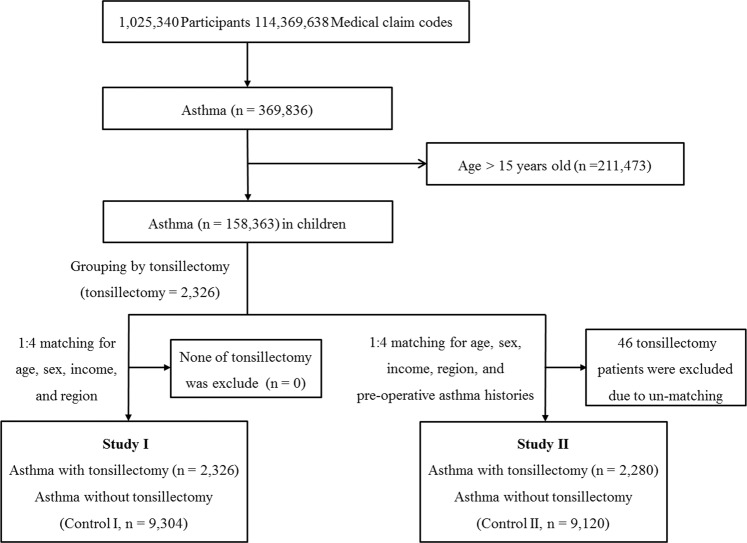
A schematic illustration of the participant selection process that was used in the present study. Of a total of 1,125,691 participants, 158,363 children (≤14 years old) had a history of asthma from 2002 through 2013. Among them, 2,326 participants underwent tonsillectomy from 2003 through 2010. The tonsillectomy participants were matched 1:4 with a control I group of participants who did not undergo tonsillectomy for age, sex, income, and region (study I) and a control II group for age, sex, income, region, and preoperative asthma history (study II). In study II, unmatched tonsillectomy participants were excluded (n = 46). Finally, 2,326 tonsillectomy participants and 9,304 control I participants were included in study I, and 2,280 tonsillectomy participants and 9,120 control II participants were included in study II.

Of 1,125,691 patients with 114,369,638 medical claim codes, we included participants with a history of asthma (ICD-10: J 45) and status asthmaticus (J 46) who were treated ≥1 time and who were treated with corticosteroids, steroid inhalers, long-acting muscarinic antagonists (LAMA), leukotriene receptor antagonists (LTRA), and xanthine (n = 369,836) from 2002 through 2013, as described in previous study^29^. Among them, we excluded adolescents and adults (age >15 years old, n = 211,473). Among the children with a history of asthma (n = 158,363), we selected participants who underwent tonsillectomy (claim code: Q2300) from 2003 through 2010 (n = 2,326). The tonsillectomy participants were matched 1:4 with participants (control group) who had not undergone tonsillectomy from 2002 through 2013 among this cohort. The matched participants were processed for age group, sex, income group, and region of residence. To prevent selection bias when selecting the matched participants, the control I group participants were sorted using a random number order, and they were then selected from top to bottom. No one was unmatched. Finally, 1:4 matching resulted in the inclusion of 2,326 asthma participants who underwent tonsillectomy and 9,304 asthma participants who did not undergo tonsillectomy (control I) (Fig. 1).

#### Study II

The participants were selected according to the same methods described for study I. However, the tonsillectomy participants were 1:4 matched with the control II group for age, group, sex, income group, region of residence, and the number of preoperative diagnoses of asthma (J 45) during one year. For example, one participant who underwent tonsillectomy in 2006 and who visited an outpatient clinic three times for asthma in 2005 was matched with 4 participants who did not undergo tonsillectomy and who visited an outpatient clinic the same number of times in 2005. To prevent selection bias, the control II group participants were sorted using a new random number order, and they were then selected from top to bottom. The tonsillectomy participants for whom we could not identify enough matching participants were excluded (n = 46). Finally, 1:4 matching resulted in the inclusion of 2,280 asthma participants who underwent tonsillectomy and 9,120 asthma participants who did not undergo tonsillectomy (control II) (Fig. 1).

#### Variables

The age groups were classified using 5-year intervals: 0–4, 5–9, and 10–14 years old. A total of three age groups were designated. The income groups were initially divided into 41 classes (one health aid class, 20 self-employment health insurance classes, and 20 employment health insurance classes). These groups were recategorized into 11 classes (class 1 [lowest income]-11 [highest income]). Region of residence was divided into 16 areas according to administrative district. These regions were regrouped into urban (Seoul, Busan, Daegu, Incheon, Gwangju, Daejeon, and Ulsan) and rural (Gyeonggi, Gangwon, Chungcheongbuk, Chungcheongnam, Jeollabuk, Jeollanam, Gyeongsangbuk, Gyeongsangnam, and Jeju) areas.

We analyzed the number of visits to an outpatient clinic for asthma (J 45) and status asthmaticus (J 46) using the ICD-10 codes. Additionally, we evaluated the number of admissions or visits to an emergency room (ER) for asthma (J 45 or J 46). The number of visits to a clinic or hospital for asthma, status asthmaticus, or admission (including ER visits) were counted for each year. Preoperative visits were counted for one year, and postoperative visits were counted for 3 years (e.g., postoperative 1 year, 2 years, and 3 years).

### Statistical analyses

An equivalence test was used to compare the number of visits for asthma, status asthmaticus, and admission between the asthma with tonsillectomy group and the asthma without tonsillectomy group. The null hypothesis was that visits during the follow-up period would not be the same between the tonsillectomy and control groups. In a previous study, the risk difference for asthma between the tonsillectomy and control groups was −0.05 episodes per year^15^. Therefore, the margin of equivalence of difference (tonsillectomy - control group) was set to −0.05 to 0.05 in this study. For the equivalence test, a 95% confidence interval (CI) for a difference <0.1 was considered to indicate statistical significance.

For the subgroup analyses, the participants were divided into 2 groups: rare preoperative asthma (<2 times a year) and frequent preoperative asthma (≥2 times a year); 81.2% of participants had a diagnosis of preoperative asthma <2 times per year.

Because the equivalence test only exhibits whether the history of asthma is different or not, we performed repeated measures ANOVA for tonsillectomy according to yearly changes in asthma, status asthmaticus, and admission. In this analysis, P values less than 0.05 were considered to indicate statistical significance. The results were statistically analyzed using SPSS v. 22.0 (IBM, Armonk, NY, USA).

## Supplementary information

S1 table
