# Supplementary material for: Tonsillectomy does not reduce asthma in children: A longitudinal follow-up study using a national sample cohort
Source: Sci Rep. 2019 Sep 16;9:13382. doi: 10.1038/s41598-019-49825-3 (PMC6746861; doi:10.1038/s41598-019-49825-3)
Supplement: Supplementary file 1 — S1 table [file 41598_2019_49825_MOESM1_ESM.docx]

**Tonsillectomy does not reduce asthma in children: A longitudinal follow-up study using a national sample cohort**

So Young Kim^1^, Dong Jun Oh^2^, Hyo Geun Choi^3,4*^

^1^Department of Otorhinolaryngology-Head & Neck Surgery, CHA Bundang Medical Center, CHA University, Seongnam, Korea

^2^Department of Internal medicine, Asan Medical Center, University of Ulsan College of Medicine, Seoul

^3^Department of Otorhinolaryngology-Head & Neck Surgery, Hallym University College of Medicine, Anyang, Korea

^4^Hallym Data Science Laboratory, Hallym University College of Medicine, Anyang, Republic of Korea

*Correspondence: [pupen@naver.com](mailto:pupen@naver.com)

**S1 table**. Subgroup analysis of pre-operative and post-operative asthma between tonsillectomy and control group according to preoperative asthma frequency in Study II (very high pre-operative asthma group)

|  | Tonsillectomy  (mean, SD) | Control II  (mean, SD) | 95% CI of difference | P-value of Independent T-test | P-value of repeated measured ANOVA |
| --- | --- | --- | --- | --- | --- |
| **Very high pre-operative asthma (≥ 5 times a year, n = 660)** | | | | | |
| Pre-op asthma | 6.90 ± 2.55 | 6.90 ± 2.54 | -0.49 to 0.49 | 1.000 | 0.525 |
| Post-op 1 year asthma | 2.03 ± 3.98 | 2.48 ± 4.19 | -0.34 to 1.24 | 0.266 |  |
| Post-op 2 year asthma | 1.22 ± 2.22 | 1.70 ± 3.28 | -0.12 to 1.07 | 0.115 |  |
| Post-op 3 year asthma | 1.04 ± 2.27 | 1.28 ± 2.73 | -0.27 to 0.74 | 0.354 |  |

* Statistical significance at P < 0.05

SD: Standard deviation

CI: Confidence interval

Difference: Control group – Tonsillectomy group

Repeated measured ANOVA using Greenhouse-Geisser correction
